# A Systematic Review and Meta-Analysis of the *Campylobacter* spp. Prevalence and Concentration in Household Pets and Petting Zoo Animals for Use in Exposure Assessments

**DOI:** 10.1371/journal.pone.0144976

**Published:** 2015-12-18

**Authors:** Katarina D. M. Pintar, Tanya Christidis, M. Kate Thomas, Maureen Anderson, Andrea Nesbitt, Jessica Keithlin, Barbara Marshall, Frank Pollari

**Affiliations:** 1 Centre for Foodborne, Environmental and Zoonotic Infectious Diseases, Public Health Agency of Canada, Ottawa, Ontario, Canada; 2 Ontario Ministry of Agriculture, Food and Rural Affairs, Guelph, Ontario, Canada; 3 Centre for Public Health and Zoonoses, University of Guelph, Guelph, Ontario, Canada; GI Lab, UNITED STATES

## Abstract

Animal contact is a potential transmission route for campylobacteriosis, and both domestic household pet and petting zoo exposures have been identified as potential sources of exposure. Research has typically focussed on the prevalence, concentration, and transmission of zoonoses from farm animals to humans, yet there are gaps in our understanding of these factors among animals in contact with the public who don’t live on or visit farms. This study aims to quantify, through a systematic review and meta-analysis, the prevalence and concentration of *Campylobacter* carriage in household pets and petting zoo animals. Four databases were accessed for the systematic review (PubMed, CAB direct, ProQuest, and Web of Science) for papers published in English from 1992–2012, and studies were included if they examined the animal population of interest, assessed prevalence or concentration with fecal, hair coat, oral, or urine exposure routes (although only articles that examined fecal routes were found), and if the research was based in Canada, USA, Europe, Australia, and New Zealand. Studies were reviewed for qualitative synthesis and meta-analysis by two reviewers, compiled into a database, and relevant studies were used to create a weighted mean prevalence value. There were insufficient data to run a meta-analysis of concentration values, a noted study limitation. The mean prevalence of *Campylobacter* in petting zoo animals is 6.5% based on 7 studies, and in household pets the mean is 24.7% based on 34 studies. Our estimated concentration values were: 7.65x10^3^cfu/g for petting zoo animals, and 2.9x10^5^cfu/g for household pets. These results indicate that *Campylobacter* prevalence and concentration are lower in petting zoo animals compared with household pets and that both of these animal sources have a lower prevalence compared with farm animals that do not come into contact with the public. There is a lack of studies on *Campylobacter* in petting zoos and/or fair animals in Canada and abroad. Within this literature, knowledge gaps were identified, and include: a lack of concentration data reported in the literature for *Campylobacter* spp. in animal feces, a distinction between ill and diarrheic pets in the reported studies, noted differences in shedding and concentrations for various subtypes of *Campylobacter*, and consistent reporting between studies.

## Introduction

Zoonoses can be transmitted from food [1–6], water [7,8], and animal contact [9–16]. *Campylobacter* is a zoonotic organism that contributes significantly to the burden of enteric illness globally and in Canada, as Campylobacteriosis is the leading cause of bacterial acute gastrointestinal disease [17,18]. Not all strains of *Campylobacter* are consistently attributed to human infections. *Campylobacter upsaliensis* is a frequently isolated strain in sick and healthy animals [19–22] but does not necessarily result in human illness [23–25]. It is thus worthwhile to consider *Campylobacter* transmission risks while excluding the prevalence and concentration of *Campylobacter upsaliensis* until we know more about its prevalence in human cases

Outbreaks of enteric diseases have been associated with petting zoos [26,27] and household pets [28–31], though many gaps in our understanding of the public health risks associated with these transmission routes remain, as most existing surveillance programs do not consider animals, and if they do, focus on the risks associated with farm animals and rural exposures [9–14,32,33]. Physicians may underestimate the importance of pet contact as a source of zoonotic disease, especially for immunocompromised or high-risk patients [34]. As urban populations grow and interactions with farm animals become less frequent [26], the relative importance of zoonotic disease transmission from household pets and petting zoo animals thus increases [33,35].

Petting zoo animals and household pets are not captured in routine public health surveillance. There are many elements of petting zoo design and associated behaviours of the visiting public that can be quantified in greater detail to enhance operator and visitor education and operation guidelines to manage risks to the public [36]. Likewise, households with pets produce numerous opportunities for pathogen exposure [34], and limited knowledge of the associated risks is a cause for concern and an opportunity for intervention [37].

It is valuable to review and synthesize the available literature to inform risk assessments, especially to bring greater awareness to the risks associated with petting zoo animal exposures and highlight where there are existing knowledge and data gaps. Quantifying a representative, weighted mean prevalence and concentration of *Campylobacter* in commonly contacted animals in Canada, the United States, Europe, Australia, and New Zealand has not yet been done. This study contributes to the body of evidence that is being gathered to inform our understanding of the multiple routes of exposure involved in the overall burden of campylobacteriosis. Efforts to systematically capture the available evidence are critical to inform public health risk management and decision-making, and prioritization of interventions.

The purpose of this study was to quantify the prevalence and concentration of *Campylobacter* carriage in animals to which the public are exposed: household pets and petting zoo animals. The question being addressed is: How often and how heavily are the feces, urine, hair coat or mouths of household pets and farm animals, with which the general public may come in contact, contaminated with *Campylobacter*? The results of this meta-analysis will be used to inform exposure models for animal contact to further inform source attribution studies of campylobacteriosis.

## Materials and Methods

Systematic review and meta-analysis were used to estimate *Campylobacter* spp. prevalence in household pets and petting zoo animals and to identify data gaps.

### Systematic review search strategy and selection criteria

Literature searches for published studies were conducted on February 16, 2012 of the following databases/data platforms (Table 1): PubMed, CAB direct, ProQuest, and Web of Science. Searches retrieved literature regarding enteropathogen prevalence and concentration in household pets and animals that are in contact with the public. All searches were for English language publications only. Publication types included journal articles and outbreak reports. No review protocol exists.

**Table 1:** Electronic search strategies for research databases and search results.

Database	Petting Zoo Hits	Household Pets Hits	Search strategy
CAB Direct	202	71	“All Fields”
ProQuest	68	24	“All Fields, no full text Books, conference papers & proceedings, dissertations & theses, encyclopaedias & reference works, government & official publications, reports, scholarly journals”
PubMed	496	269	“Titles/Abstract”
Web of Science	837	497	“Topic”

The search started as a scoping review of papers examining the prevalence and concentration of three enteropathogens in household pets and petting zoo animals. Two different searches were performed: *Cryptosporidium*, *Giardia*, and *Campylobacter* in petting zoo animals (S1 Table), and *Cryptosporidium*, *Giardia*, and *Campylobacter* in household pets (S2 Table). This search was restricted to Canada, USA, Europe, Australia, and New Zealand, the years 1992–2012, animal search terms (adapted from the literature [36,38]), and other search terms including fecal, hair coat, oral, or urinary exposure routes.

Inclusion was based on the following criteria: published in English, published between 1992 and 2012, reported naturally occurring disease/carriage, and investigated a relevant pathogen in animals in contact with the public (animals from petting zoos, fairs, or shows; production animals in public displays or open farms) or animals kept as a household pet.

Exclusion criteria specified that articles not include experimental infection/carriage, foodborne contamination from the species in question, or waterborne exposure without direct testing of animals/animal excretions. Articles were also excluded if they only investigated laboratory animals, feral animals, research colonies of dogs or cats from commercial facilities, or production animals: major farm species (cattle, swine, poultry, small ruminants), and purpose-bred animals with no contact with the general public (race horses, racing greyhounds). Finally, papers that were test validation studies or treatment evaluations studies were also excluded.

All search results were imported into Refworks reference software [39] for duplicate removal and further screening as per exclusion criteria. Attempts were then made to retrieve copies of all remaining references for review. Additional references were added via hand searches of the reference lists of the selected papers, searching through the papers that cited the selected articles (before 2012), and through branching by examining the reference lists of relevant reports, primary and review articles, and Google searches for the full text of some publications for sources that matched the original inclusion/exclusion study criteria. Reports that contained information pertinent to the research questions were then entered in the database. Some papers were removed in the final database because they could not be accessed by the reviewer, they were discovered to be additional duplicates or outside of the time frame of intent, citations were for book reviews, or they could not be retrieved electronically and hard copies were not received before the project deadline.

### Data extraction for qualitative synthesis

A database was created to include prevalence and concentration values along with other study information (S3 and S4 Tables). Additional extraction variables included: the animals studied; the different pathogens investigated; whether detection was quantitative or qualitative; whether detection was based on culture (for bacteria) or microscopy (for parasites) or molecular techniques (including PCR on DNA extracted from raw samples or antigen testing on samples); whether molecular (i.e. DNA-based/PCR) techniques were used to characterize strains/isolates detected by other means (e.g. culture/microscopy); whether samples were pooled (potentially from multiple animals) or collected from the environment (observed freshly passed feces from single animals were not considered environmental samples); whether carriage of pathogens was fecal, oral, urinary or found on the skin/haircoat; and whether the number of cases of human illness due to pathogens were included in the report, if any. Quality criteria recorded for studies included: location of the study (Canada, USA, Europe, Australia, or New Zealand); whether the pathogen detection technique (culture, microscopy or molecular) was either described, referenced or stated as “done according to manufacturer’s instructions (if the technique was only named with no further description or reference this was recorded as not described); whether pathogen detection (or characterization) was done at a government/state lab; whether the strains/species detected in animals were also detected in human cases who had been in contact with the animal, and if not, whether the strains/species detected in animals were reported to infect humans; and the source of animal samples. i.e. the population investigated.

### Data extraction for meta-analysis

Data for *Campylobacter* prevalence and concentration were extracted from the studies identified through the systematic review of the literature and included in the study database independently by a single trained reviewer and validated by a second. The prevalence of fecal, oral, urinary or skin/haircoat *Campylobacter* in the animal studies was defined as the proportion of positive samples collected from the specific animal population. Data were extracted for animals of interest (S1 and S2 Tables) that were either household pets or animals that could come in contact with the public, such as at a fair/zoo/exhibit.

Among the household pet population, prevalence values for diarrheic animals were included, a potential source of bias. Studies in which the sample population size was not clearly reported and studies reporting farm-level proportions were excluded from data extraction. In studies reporting prevalence estimated from repeated measures, the most clinically significant results were extracted. Data were extracted for *Campylobacter* detected by culture and isolation techniques or by molecular (PCR) methodologies. In some cases, studies relied exclusively on culture-based detection for *Campylobacter* spp.—those studies were included. In other cases, studies relied on a combination of molecular and/or culture-based detection. Wherever possible, we extracted the prevalence estimates that were species specific, which was most often based on molecular methods. Data were extracted for the prevalence and concentration of total *Campylobacter* spp. but when specified, estimates of *Campylobacter upsaliensis* were excluded. *Campylobacter upsaliensis* was excluded when possible as it is a frequently isolated strain of *Campylobacter* in animals yet its public health significance is currently not well-understood [19–22] Including *C*. *upsaliensis* in the prevalence estimates could result in an overestimation of risk [23–25]. For this reason, two prevalence values were produced: one overall prevalence value (which excludes *Campylobacter upsaliensis* where possible) and a revised prevalence that includes studies where *Campylobacter upsaliensis* is identified but cannot be excluded because of a lack of available data.

### Data analysis

The overall prevalence of *Campylobacter* in household pets and petting zoo species was estimated by meta-analysis as a weighted mean, and run a second time incorporating *Campylobacter upsaliensis* prevalence. The weighted mean prevalence was computed from the proportion of positive animals and the number of animals sampled in each study, allowing some data points to contribute more to the overall mean than others (based on sample size, in this case) [40]. Study-specific effects are variations in study conditions that cannot be quantified or explicitly characterized, which may result in variations in effect size [41–43]. By calculating a weighted mean through a random effects model the study-specific variation in the effects between and within studies can be controlled [42,43]. Given the *a priori* assumption that the study populations would comprise significant differences, random effects models were constructed [44]. Prevalence estimates for each study and the corresponding summary estimate were generated using the Metaprop command in Stata v12 (StataCorp LP, College Station, Texas). The model was fit to a DerSimonian-Laird random effects model that assumes heterogeneity between the studies and outcome values. The assumption behind random effects models is that there is between- and within-study variability in the results, whereas fixed effects models are fit to data where variance is unlikely and occurs only as a result of sampling error. It was hypothesized that the prevalence of *Campylobacter* in our populations of interest would vary widely thus a random effects model was used. In order to analyse model sensitivity the meta-analysis was run a second time substituting a random effects model for a fixed effects model. Variance was stabilized using the Freeman-Tukey arcsin transformation and confidence intervals were calculated with score (Wilson) methods. In addition, a measure of heterogeneity representing the proportion of total variation that was due to between study variation in the estimation of the weighted mean was reported (I^2^) [40]. Potential sources of heterogeneity and selection bias were addressed with sub-models within the meta-analysis. The effect of nationality, animal species, and diarrheic populations were examined.

Pathogen concentration values were extracted when possible for the animal populations of interest. Limited data prevented the development of a meta-analysis of estimates for household pets and petting zoo animals.

## Results

### Characteristics of the articles included in the systematic review

**Fig 1:**
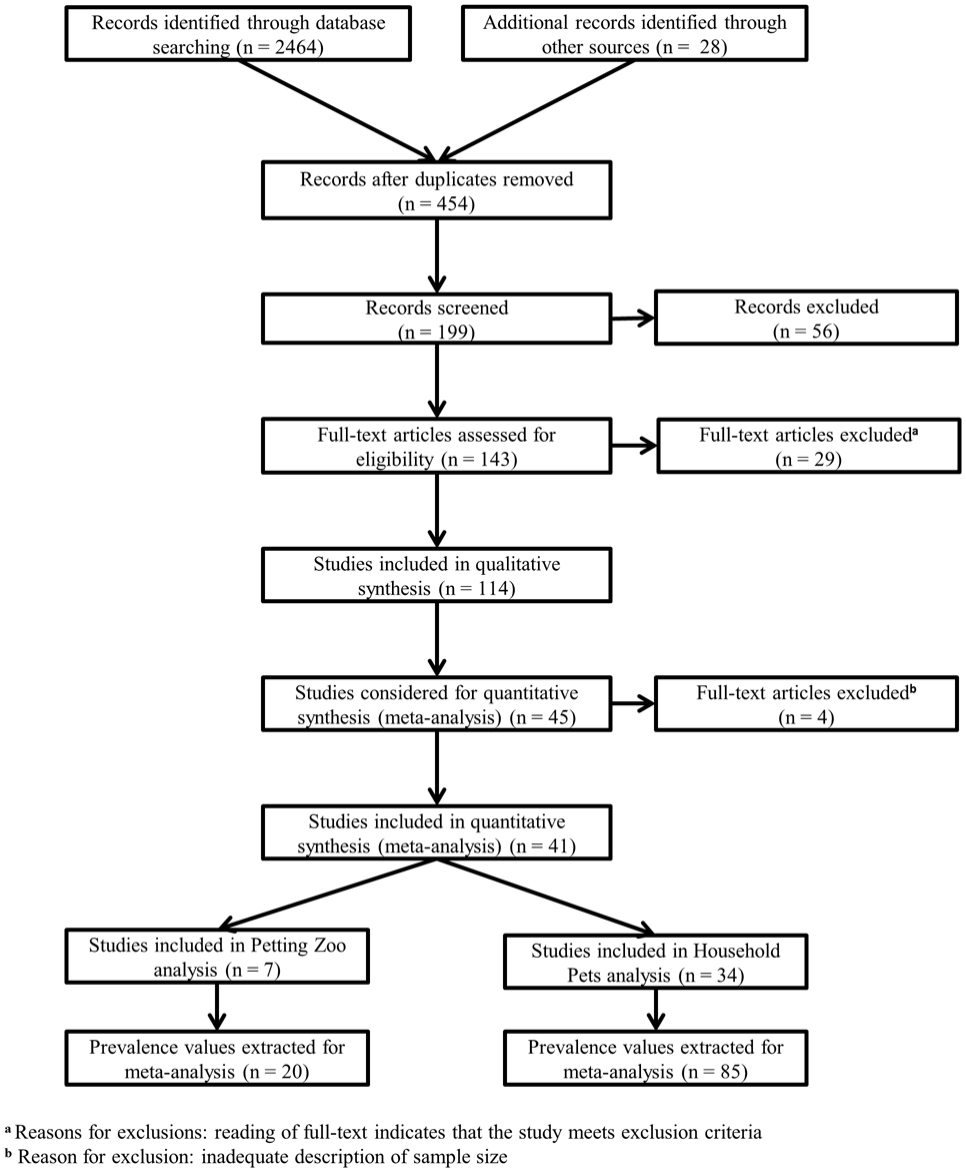
Flow diagram of *Campylobacter*, *Cryptosporidium*, and *Giardia* studies screened, assessed, included, and excluded.

The searches resulted in the identification of 2464 studies that examined *Campylobacter*, *Cryptosporidium*, and *Giardia* of which 2038 duplicates were removed (Fig 1), resulting in 426 records. An additional 28 records were found through branching (from reference lists or citation lists). The abstracts of these 454 records were assessed when available, and 199 were retained and screened for eligibility, of which 56 were excluded. Full-text articles were sought for the remaining 143 records and of these 114 were selected for inclusion in the study database (S3 and S4 Tables). These studies were assessed for inclusion in the *Campylobacter* meta-analysis and 45 were initially selected. After further analysis of the studies, 4 were excluded and only 41 were selected.

A total of 114 papers were included based on the results of the systematic review, which are described in the appendix (S3 and S4 Tables). Of these, 50 of the papers were from the USA [19–22,45–64], 26 were from the UK [16,25,65–89], 19 were from other European countries [15,90–99], 15 were from Canada [100–119], two were from Australia [120,121], and one was from New Zealand [122] (S4 Table). The populations investigated were most frequently veterinary patients, of which there were 45 studies, and animals in contact with the public, which were the population of interest in 44 studies. Following these, 22 studies examined household pets, 15 examined shelter animals, and three studies each examined laboratory submissions, kennels, and breeding operations. The most frequently studied animals were household pets (dogs and cats), with dogs appearing in 55 of the studies and cats in 44 of the studies. The most frequently studied petting zoo animals were cattle, which were included in 28 of the studies, followed by 26 studies of equids, 24 studies of sheep, 22 studies of goats and 18 studies of pigs. The research was supported from a state or government lab in 21 of the papers. Forty-six of the papers studied *Giardia*, 45 studied *Campylobacter*, and 33 studied *Cryptosporidium*. Although the search was limited to these pathogens, many articles also examined other pathogens, such as pathogenic *E*. *coli* (Shiga toxin-producing *E*. *coli* [STEC] or Verocytotoxin-producing *E*. *coli* [VTEC]) in 36 of the papers and *Salmonella* in 28 of the papers.

All 114 selected papers examined fecal carriage of pathogens as the animal outcome of interest. Twenty-five of the studies examined human illness as an outcome and 27 used pooled environmental samples. The laboratory technique of culture/microscopy was used in 102 studies followed by molecular characterization in 57 studies, and molecular detection methods in 31 studies. Similar strains within the animals and humans studied were tested in two of the studies, but more commonly no zoonotic strains/species were identified in 42 studies.

### Characteristics of the articles included in the meta-analysis

The 45 articles in the database that examined *Campylobacter* were considered for the meta-analysis. The research papers were most frequently from Europe (n = 22/45), the United States (n = 15/45), followed by Canada (n = 4/45) and the United Kingdom (n = 4/45). Four of these papers were excluded from the meta analysis for insufficient reporting of the sample size [70,85,92,123], resulting in 41 papers being considered for the meta-analysis. Of these, 7/41 examined petting zoo animals, and 34/41 considered household pets.

From the household pet papers, dogs (n = 30/34) were studied more frequently than cats (n = 18/34) and among the petting zoo papers, the most frequently examined animal was cattle (n = 5/7) followed by equids (n = 3/7). All papers examined the fecal transmission of *Campylobacter* to measure prevalence and concentration and no other routes were considered in the papers. The population under investigation was most frequently veterinary patients (n = 26/41), followed by household pets (n = 11/41) and animals in contact with the public (n = 10/41).

### Findings from the meta-analysis of prevalence values

For the petting zoos meta-analysis 20 prevalence values were extracted and included from the 7 papers (Fig 1; S3 Table). One of these prevalence values was from a population of diarrheic animals [77]. The weighted mean prevalence of *Campylobacter* in petting zoo animal populations was estimated to be 6.5% (95% CI: 4.1–9.4) (Table 2; S5 Table). Subgroup meta-analyses were performed for animal species, country, and geographic region (Table 2; S1 Fig).

**Table 2:** Subgroup meta-analysis for studies reporting the prevalence of *Campylobacter* in petting zoos.

	Number of prevalence value inputs ^a^	Sample size	Weighted mean estimate	Confidence Interval (95%)	I^2^ (%)	p-value ^b^
Overall ^c^	20	5778	0.07	(0.04–0.09)	79.9%	<0.01
**Subgroup analysis**						
**By Country**						
Netherlands	5	4833	0.12	(0.08–0.17)	89.9%	<0.01
USA	10	309	0.03	(0.00–0.09)	67.9%	<0.01
UK	3	588	0.07	(0.05–0.09)	0.0%	0.64
Germany	2	48	0.18	(0.08–0.31)	0.0%	0.36
**By Region**						
Europe	10	5469	0.10	(0.07–0.13)	83.3%	<0.01
North America	10	309	0.03	(0.00–0.09)	67.9%	<0.01
**By Species**						
Multiple species	3	2383	0.34	(0.02–0.78)	90.3%	<0.01
Cattle	7	2606	0.10	(0.06–0.15)	79.6%	<0.01
Sheep	2	77	0.09	(0.00–0.44)	90.1%	<0.01
Swine	1	56	0.01	(0.00–0.06)	.	.
Horses	4	613	0.07	(0.05–0.09)	0.0%	0.67
Goats	2	42	0.10	(0.02–0.22)	0.0%	0.35
Llama	1	1	0.50	(0.00–1.00)	.	.

^a^ prevalence values and sample sizes for each study provided in S3 Table.

^b^ p-values accompany I^2^ values and test for heterogeneity.

^c^ The sensitivity analysis, which used a fixed effects model, resulted in a prevalence value of 0.07 (95% CI 0.07–0.08).

The household pets literature search resulted in the inclusion of 34 publications that reported on *Campylobacter* prevalence (S3 Table). From these 34 papers, 85 prevalence values were extracted. The resulting weighted mean prevalence of *Campylobacter* for household pets was 24.7% (95% CI: 19.8–29.9) (Table 3; S2 Fig). In cases where the studies differentiated between subtypes, *Campylobacter upsaliensis* was excluded, which is why sub-models with only one input may have valid prevalence and revised prevalence values. When *Campylobacter upsaliensis* prevalence was included, the prevalence was 34.0% (95% CI: 11.1–17.6) (Table 3; S3 Fig). Subgroup meta-analyses were performed using the prevalence value and the revised prevalence value (excluding *Campylobacter upsaliensis*) assessing subgroups of animal species, country, region, diarrheic status, and the source of the animals under investigation (Table 3; S2 and S3 Figs). A one-tailed paired t-test was performed on input values for prevalence and revised prevalence and a significant difference between these two groups of prevalence values was found (p < 0.001).

**Table 3:** Subgroup meta-analysis for studies reporting the prevalence of *Campylobacter* in household pets.

		Prevalence ^a^					Revised Prevalence ^b^					
	Number of prevalence value inputs	Sample size	Weighted mean estimate	Confidence Interval (95%)	I^2^ (%)	p-value ^c^	Number of prevalence value inputs	Sample size	Weighted mean estimate	Confidence Interval (95%)	I^2^ (%)	p-value ^c^
Overall ^d^ ^,^ ^e^	85	8730	0.25	(0.20–0.30)	96.4%	<0.01	40	6071	0.34	(0.28–0.41)	96.5%	<0.01
**Subgroup analysis**												
**By country**												
UK	24	2213	0.35	(0.27–0.45)	94.2%	<0.01	6	955	0.47	(0.33–0.61)	94.7	<0.01
Australia	6	276	0.06	(0.01–0.15)	71.1%	<0.01	4	215	0.09	(0.02–0.19)	67.3	0.03
USA	23	1640	0.10	(0.05–0.15)	91.0%	<0.01	3	380	0.25	(0.02–0.60)	97.3	<0.01
Switzerland	4	1627	0.29	(0.17–0.42)	96.7%	<0.01	4	1627	0.29	(0.17–0.42)	96.7	<0.01
Spain	1	290	0.35	(0.30–0.41)	.	.	1	290	0.35	(0.30–0.41)	.	.
Canada	5	537	0.36	(0.03–0.80)	99.0%	<0.01	3	195	0.70	(0.33–0.97)	96.6	<0.01
Sweden	2	91	0.65	(0.35–0.90)	61.6%	0.11	0	0	.	.	.	.
Denmark	3	480	0.34	(0.01–0.81)	98.6%	<0.01	3	480	0.34	(0.01–0.81)	98.6	<0.01
Germany	4	307	0.43	(0.37–0.50)	14.8%	0.32	4	307	0.43	(0.37–0.50)	14.9	0.32
Belgium	2	87	0.48	(0.32–0.64)	57.2%	0.13	2	87	0.47	(0.37–0.58)	89.1	<0.01
Italy	4	274	0.32	(0.21–0.45)	75.3%	<0.01	4	274	0.32	(0.21–0.45)	75.3	0.01
Norway	4	557	0.21	(0.17–0.26)	42.0%	0.16	4	927	0.21	(0.17–0.26)	42.0	0.16
Czech Rep.	3	351	0.19	(0.13–0.25)	36.6%	0.21	2	334	0.20	(0.16–0.24)	89.1	<0.01
**By region**												
Europe	51	6272	0.34	(0.29–0.40)	94.8%	<0.01	30	5281	0.35	(0.29–0.42)	95.7	<0.01
Austral./NZ	6	276	0.06	(0.01–0.15)	71.1%	<0.01	4	215	0.09	(0.02–0.19)	67.3	0.03
North America	28	2177	0.13	(0.07–0.22)	96.1%	<0.01	6	575	0.47	(0.15–0.81)	98.5	<0.01
**By species**												
Dogs	51	6039	0.31	(0.25–0.38)	96.8%	<0.01	25	4406	0.40	(0.32–0.49)	97.0	<0.01
Cats	33	2565	0.15	(0.09–0.23)	94.5%	<0.01	14	1539	0.25	(0.15–0.36)	94.5	<0.01
Cats & Dogs	1	126	0.15	(0.10–0.22)	.	.	1	126	0.15	(0.10–0.22)	.	.
**By source**												
Shelter	12	855	0.29	(0.13–0.48)	96.7%	<0.01	5	542	0.30	(0.09–0.57)	97.2	<0.01
Household pets	25	1643	0.20	(0.08–0.35)	97.2%	<0.01	4	679	0.51	(0.24–0.78)	97.8	<0.01
Kennel	7	810	0.28	(0.14–0.45)	95.6%	<0.01	4	311	0.41	(0.17–0.68)	95.4	<0.01
Clinic	39	5051	0.27	(0.21–0.33)	95.5%	<0.01	27	4539	0.31	(0.25–0.38)	95.2	<0.01
Clinic & Shelter	2	371	0.08	(0.04–0.12)	56.7%	0.13	0	0	.	.	.	.
**By diarrheic status**												
Diarrheic	28	2140	0.26	(0.17–0.36)	95.3%	<0.01	14	1479	0.31	(0.18–0.45)	96.6	<0.01
Non-diarrheic	56	6438	0.25	(0.19–0.31)	96.5%	<0.01	26	4592	0.36	(0.29–0.44)	95.8	<0.01
Mixed pop.	1	152	0.05	(0.02–0.10)	.	.	0	0	.	.	.	.

^a^ prevalence values and sample sizes for each study provided in S4 Table.

^b^ Includes *Campylobacter upsaliensis*.

^c^ p-values accompany I^2^ values and test for heterogeneity.

^d^ these values are significantly different (p < 0.001) based on a one-tailed paired t-test of the raw values used to produce the weighted means.

^e^ The sensitivity analysis, which used a fixed effects model, resulted in a prevalence value of 0.11 (95% CI 0.11–0.12) and a revised prevalence of 0.34 (95% CI 0.33–0.35).

There are I^2^ values in the study that are high and some that are 0%. Although lower I^2^ values are preferable, a value of 0% heterogeneity is not ideal. These values are a reflection of having few study inputs and do not reflect a lack of variation between input values. Despite these results, a fixed effects model would be an inappropriate choice, as prevalence values are expected to vary. When fixed effects models were run, the weighted mean estimate for petting zoo animals did not change although the confidence interval narrowed (random effects: 0.07 (95% CI 0.04–0.09) fixed effects: 0.07 (95% CI 0.07–0.08). The weighted mean estimate for household pets was higher when a random effects model was used (0.25 (95% CI 0.20–0.30)) compared to the fixed effects model (0.11 (95% CI 0.11–0.12)). Further, some summary estimates produced I^2^ values that indicate high heterogeneity (I^2^ = 96.4% for household pets and 79.9% for petting zoo animals) suggesting that there is considerable heterogeneity in the study inputs. These high I^2^ values indicate that a random effects model was a better choice than a fixed effects model, as the random effects model is more appropriate for heterogeneous estimates.

### Findings from the review of *Campylobacter* concentration values

The mean fecal concentration of *Campylobacter* in the petting zoo animals was based on one study which reported *Campylobacter jejuni* in cattle at a farm that is visited by the public [93].The values reported in this study were a range (3.0x10^2^-1.5x10^4^), so the value was estimated to be the average of the low and high values (7.65x10^3^) (Table 4). Two studies reported three concentration values for fecal *Campylobacter* levels in household pets. One paper included two concentration values from 60 fecal samples: for *Campylobacter jejuni* (2.9x10^5^ (range: 1.6x10^4^–2.3x10^6^) and *Campylobacter upsaliensis* (8.6x10^5^ (range: 6.0x10^3^–1.3x10^7^)) [105]. The other study reported a range (10^3^–10^8^) from 135 fecal samples, which included *Campylobacter upsaliensis* [102]. The two values that included *Campylobacter upsaliensis* and were excluded, leaving only one study that reported the concentration of *Campylobacter jejuni* which was the value used to approximate *Campylobacter* spp. in household pets (Table 4).

**Table 4:** Concentration of *Campylobacter* in feces of petting zoo and household pet populations as reported in February 2012 literature search results.

	Household pets	Petting zoo species
Studies included	1	1
Concentration of *Campylobacter* spp. (cfu/g feces)	2.9x10^5^	7.65x10^3^ ^a^
Estimated Range	1.6x10^4^–2.3x10^6^	3.0x10^2^–1.5x10^4^

^a^ Average of the range values.

## Discussion

In this study, a rigorous, comprehensive, and transparent approach was taken to identify publications reporting the prevalence and concentration of *Campylobacter* in household pet and petting zoo populations in Canada, the United States, Europe, Australia, and New Zealand. This study is a necessary step in identifying and quantifying the risk of *Campylobacter* exposure associated with household pets and petting zoos as well as identifying data gaps to encourage future research directions. Following an approach developed in Europe[124], a larger study was initiated to quantify the exposure of Ontarians to *Campylobacter* from 13 specific exposure routes of campylobacteriosis: foodborne (beef, pork, chicken, seafood/fish, vegetables, fruits), waterborne (drinking water, recreational water), and animal contact (household pets, petting zoos, visiting a farm, living on a farm) [125].

The results of the meta-analyses are an important first step in quantifying *Campylobacter* carriage in household pets and petting zoos, which is not measured as part of public health surveillance in Canada. Although the high heterogeneity associated with the summary estimates (I^2^ values of 96.4% and 79.9% for household pets and petting zoos respectively) introduces limitations into their interpretation and use, the summary estimate is a more representative estimate for *Campylobacter* prevalence in household pets and petting zoo animals compared to estimates from individual studies or the arithmetic mean of multiple studies. A sensitivity analysis of the results was performed by running inputs with a fixed effects model. The results of the fixed effects model were relatively similar to the random effects models for the petting zoos model and the revised household pets prevalence, but differed for the household pets prevalence. The fixed effects models resulted in 95% Confidence Intervals that were narrower than the random effects models.

The prevalence of *Campylobacter* estimated in petting zoo animals (approximately 6.5%) when compared to FoodNet Canada data from farms, is lower than swine, dairy cattle, beef cattle, and broiler chickens prevalence values (85%, 80%, 82%, 8.4% respectively using pooled manure samples) [126]. However, the FoodNet Canada samples are collected from farm animals that are raised for food production, thus conditions that may influence *Campylobacter* carriage will be different than in petting zoo environments. A recent study from Quebec determined the prevalence of *Campylobacter* at dairy farms (72.5%) [127].

The prevalence of *Campylobacter* in household pets (approximately 24.7%) is higher than the petting zoo estimate and the prevalence reported by FoodNet Canada for broiler chickens, but lower than the prevalence reported for swine, dairy cattle, and beef cattle found by FoodNet Canada. Unlike the petting zoo estimate, the prevalence values used for the household pets estimate were adjusted to exclude *Campylobacter upsaliensis*. When this species was included, the prevalence of *Campylobacter* spp. in household pets was higher (34.0%).

Due to the high heterogeneity associated with the summary estimates for both pets and petting zoos, potential sources of heterogeneity were explored with subgroup meta-analysis The sub-models analysis also sheds light on potential sources of selection bias. Animal species are known to vary in prevalence and concentration of *Campylobacter* carriage [78,128]. Subgroup meta-analysis demonstrated that species may influence the heterogeneity observed in the summary measure. In particular, the studies containing multiple species suggest an increased prevalence of *Campylobacter*. However the limited number of studies prevents a full exploration of this observation, and is identified as an area of further research. The limited data available for other species (only one paper each for sheep, swine, goats, and llama) hindered a fulsome examination of the influence of different animal species on *Campylobacter* carriage and public health exposure risks. The literature is mixed on whether diarrheic animals differ by pathogenic carriage, making this a valuable comparison [77,129,130]. Our findings suggest that perhaps there is not a difference, however the high heterogeneity associated with both subgroup meta-analyses indicates a wide range of values is present in the literature and alternative sources of heterogeneity not identified in this study may be more influential. *Campylobacter* varies by geography and climate, [131,132] and our results found that the subgroup meta-analysis by the nationality of the study indicates that there may be some geographic differences in the prevalence of *Campylobacter* among pets and petting zoos species. Understanding the effect of geography is again limited in this study and additional research is needed to explore factors that influence within-country prevalence and could explain the heterogeneity observed in this analysis.

### Limitations and data gaps

Limiting the search to select countries may have biased our results but these papers were most likely to reflect the animal population conditions experienced in North America. Subgroup meta-analyses were used whenever there was enough data to address potential selection bias based on the country of origin, animal species, and the inclusion of diarrheic animals however limited data and high heterogeneity in the subgroup meta-analysis indicates that factors affecting the range of prevalence values seen in the literature are yet to be identified. Publication bias may have impacted the results, as unfavourable research results are typically considered to be less worthwhile for publication. However, in the studies that were captured in the review, many reported a prevalence of zero, indicating that research with negative or inconclusive findings were published in the peer-reviewed literature.

Some animals that are reported to be present in petting zoos (rabbits, donkeys, ponies, baby chicks, and llamas) [36,83,133] or household pets (rabbits) [134] were not identified in the literature that was found, and were not included in this study, representing an information gap. For animals that were included, in many cases (goats, llamas, sheep, and swine) there were not enough studies to analyse the species-level values. Investigating the level of risk in key sub-populations of animals would be worthwhile. The high degree of mixing of different species in an open farm/petting zoo setting may contribute to increased pathogen shedding among the animals [73,135,136] and young animals or clinically ill animals are often thought to be at higher risk for shedding certain pathogens [130,133,137,138].

The values for prevalence and concentration in this analysis are limited by a lack of data, which is a key finding of this study. Pathogen prevalence in animals used in public displays/exhibits is typically measured in the course of an outbreak investigation, as exhibits have already dispersed by the time the outbreak is detected. Many studies lacked a standardized reporting structure for prevalence values: the study locations, sample size (a clearly stated numerator and denominator), inclusion of diarrheic animals, and the study periods. The concentration values for both household pets and petting zoo animals could not be estimated with a meta-analysis and the estimations were each based on only one paper, representing an important information gap.

There are no available data regarding coat/skin, oral, or urinary carriage of *Campylobacter* in animals in contact with the public, which are potential routes even though carriage would likely be very low [46,93,94,139–142]. These behaviours can potentially be a significant source of pathogens via household pet contacts.

The proportion of *Campylobacter* species in each source, and the quantification of shedding are important for accurately estimating the risk to public health in a quantitative microbial risk assessment or exposure assessment ([2,143]. This study did not differentiate between subtypes of *Campylobacter* in part due to the diagnostic testing methods used in the studies included (e.g. enrichment culture, fecal antigen tests), and in part because the use of molecular subtyping of *Campylobacter*, such as MLST or CGF, is not widespread. With the increasing use of molecular detection techniques for primary detection, such as PCR, there needs to be an emphasis placed on the development of quantitative tests as well (i.e. quantitative PCR, semi-quantitative antigen tests) [144,145].

## Conclusions

This review was not intended to be exhaustive but to provide the best available estimates for prevalence and concentration of *Campylobacter* in household pets and petting zoo animals. There are likely additional studies pertinent to the project objectives that were not retrieved through the database searches or secondary searches of study references. Nonetheless, this review provides a comprehensive, relatively broad cross-section of the available literature within the inclusion/exclusion criteria set at the beginning of the project and provides a foundational summary of the literature from which to build our evidence to inform public health strategies to reduce exposure risks.

The results of the meta-analysis were used to inform a comparative exposure assessment to determine how exposure to *Campylobacter* varies between expected sources, including household pets and petting zoos. The assessment was also used to prioritize the design and implementation of programs gathering information identified as important knowledge gaps, and to inform public health interventions. The prevalence and concentration values calculated as part of this literature review and subsequent meta-analysis are crucial for the approximation of risk associated with *Campylobacter* exposure from animal contact. It is estimated that roughly 16% of all cases of *Campylobacter* infection in Canada are attributed to animal contact [146], illustrating the importance in further understanding this route of exposure to reduce the burden of enteric illness. In this study, we have provided multiple areas of research required to improve understanding of the zoonotic transmission of *Campylobacter*. Future research should quantify the concentration of *Campylobacter* in petting zoo animal and household pet feces, as well as scenarios on how *Campylobacter* exposure occurs, how contact occurs between animals and humans, and whether contact and exposure varies by animal.

## Supporting Information

S1 Fig Forest plot for meta-analysis of petting zoo animal prevalence values. (TIF)

S2 Fig Forest plot for meta-analysis of household pets prevalence values. (TIF)

S3 Fig Forest plot for meta-analysis of household pets revised prevalence values. (TIF)

S1 Table Petting Zoo Animals Search Strings. (DOCX)

S2 Table Household Pets Search Strings. (DOCX)

S3 Table Prevalence of Campylobacter in Petting Zoo Animals from Literature Review. (DOCX)

S4 Table Prevalence of Campylobacter in Household Pets from Literature Review. (DOCX)

S5 Table PRISMA Checklist. (DOC)
