# Supplementary material for: A Systematic Review and Meta-Analysis of the Campylobacter spp. Prevalence and Concentration in Household Pets and Petting Zoo Animals for Use in Exposure Assessments
Source: PLoS One. 2015 Dec 18;10(12):e0144976. doi: 10.1371/journal.pone.0144976 (PMC4684323; doi:10.1371/journal.pone.0144976)
Supplement: S1 Table — (DOCX) [file pone.0144976.s004.docx]

## S1 Table. Petting Zoo Animals Search

| **Search Function** | **Search Terms** |
| --- | --- |
| **Any of** | **enteropathogen OR enteropathogens OR enteropathogenic OR *Campylobacter* OR Campylobacteriosis OR *Cryptosporidium* OR *Cryptosporidia* OR cryptosporidiosis OR *Giardia* OR giardiosis OR giardiasis** |
| **AND Any of** | **livestock OR "farm animal" or "farm animals" OR cow OR cows OR cattle OR bovine OR calf OR calves OR sheep OR goat OR goats OR ovine OR caprine OR lamb OR lambs OR kid OR kids OR ewe OR ewes OR doe OR nannie OR nannies OR "small ruminant" OR "small ruminants" OR pig OR pigs OR piglet OR piglets OR swine OR porcine OR horse OR horses OR equine OR pony OR ponies OR zebra OR zebras OR llama OR llamas OR alpaca OR alpacas OR "south american camelid" OR "south american camelids" OR poultry OR chick OR chicks OR chicken OR chickens OR duck OR ducks OR ducklings OR goose OR geese OR goslings** |
| **AND Any of** | **fecal OR feces OR manure OR stool OR coat OR hair*coat OR fur OR fleece OR wool OR fibre OR fiber OR skin OR oral OR mouth OR saliva OR tongue OR teeth OR urine OR urinary OR urethra** |
| **Locations** | **Canada OR USA OR "United States" OR "North America" OR "United Kingdom" OR England OR Scotland OR Wales OR Ireland** |
| **Timespan** | **1992-2012** |
