# Supplementary material for: A Systematic Review and Meta-Analysis of the Campylobacter spp. Prevalence and Concentration in Household Pets and Petting Zoo Animals for Use in Exposure Assessments
Source: PLoS One. 2015 Dec 18;10(12):e0144976. doi: 10.1371/journal.pone.0144976 (PMC4684323; doi:10.1371/journal.pone.0144976)
Supplement: S2 Table — (DOCX) [file pone.0144976.s005.docx]

**S2 Table. Household Pets Search**

| Search Function | Search Terms |
| --- | --- |
| Any of | enteropathogen OR enteropathogens OR enteropathogenic OR *Campylobacter* OR Campylobacteriosis OR *Cryptosporidium* OR *Cryptosporidia* OR cryptosporidiosis OR *Giardia* OR giardiosis OR giardiasis |
| AND any of | "companion animal" OR "companion animals" OR pet OR pets OR dog OR dogs OR canine OR puppy OR puppies OR cat OR cats OR feline OR kitten OR kittens OR rabbit OR rabbits OR reptile OR reptiles OR reptilian OR lizard OR lizards OR snake OR snakes OR turtle OR turtles OR terapin OR terapins OR tortoise OR tortoises OR dragon OR dragons OR hamster OR hamsters OR "guinea pig" OR "guinea pigs" OR gerbil OR gerbils OR rat OR rats OR mouse OR mice OR rodent OR rodents OR ferret OR ferrets OR hedgehog OR hedgehogs OR chinchilla OR chinchillas OR "sugar glider" OR "sugar gliders" |
| And any of | fecal OR feces OR manure OR stool OR coat OR hair*coat OR fur OR fleece OR wool OR fibre OR fiber OR skin OR oral OR mouth OR saliva OR tongue OR teeth OR urine OR urinary OR urethra |
| Locations | Canada OR USA OR "United States" OR "North America" OR "United Kingdom" OR England OR Scotland OR Wales OR Ireland |
| Timespan | 1992-2012 |
