# Supplementary material for: A Systematic Review and Meta-Analysis of the Campylobacter spp. Prevalence and Concentration in Household Pets and Petting Zoo Animals for Use in Exposure Assessments
Source: PLoS One. 2015 Dec 18;10(12):e0144976. doi: 10.1371/journal.pone.0144976 (PMC4684323; doi:10.1371/journal.pone.0144976)
Supplement: S3 Table — (DOCX) [file pone.0144976.s006.docx]

**S3 Table.**

| Ref. | First Author, Year | Animal | Country^a^ | Region | Diarrheic population | Prevalence^b^ | % |
| --- | --- | --- | --- | --- | --- | --- | --- |
| [93] | Heuvelink, 2009 | multiple species, not specified | Netherlands | EU |  | 6/10 | 60.0% |
| [93] | Heuvelink, 2009 | multiple species, not specified | Netherlands | EU |  | 4/8 | 50.0% |
| [94] | Heuvelink, 2007 | multiple species, not specified | Netherlands | EU |  | 225/2365 | 9.5% |
| [94] | Heuvelink, 2007 | cattle | Netherlands | EU |  | 194/1341 | 14.5% |
| [94] | Heuvelink, 2007 | cattle | Netherlands | EU |  | 115/1109 | 10.4% |
| [142] | Kiang, 2006 | cattle | USA | N.AMER |  | 3/9 | 33.3% |
| [142] | Kiang, 2006 | cattle | USA | N.AMER |  | 1/14 | 7.1% |
| [147] | McNamara, 2011 | sheep | USA | N.AMER |  | 0/57 | 0.0% |
| [147] | McNamara, 2011 | swine | USA | N.AMER |  | 0/56 | 0.0% |
| [147] | McNamara, 2011 | cattle | USA | N.AMER |  | 0/50 | 0.0% |
| [147] | McNamara, 2011 | horses | USA | N.AMER |  | 0/25 | 0.0% |
| [147] | McNamara, 2011 | goats | USA | N.AMER |  | 0/14 | 0.0% |
| [147] | McNamara, 2011 | llama | USA | N.AMER |  | 0/1 | 0.0% |
| [77] | Netherwood, 1996 | horses | UK | EU | X | 29/365 | 7.9% |
| [77] | Netherwood, 1996 | horses | UK | EU |  | 5/99 | 5.1% |
| [77] | Netherwood, 1996 | horses | UK | EU |  | 8/124 | 6.5% |
| [148] | Smith, 2004 | cattle | USA | N.AMER |  | 7/23 | 30.4% |
| [148] | Smith, 2004 | cattle | USA | N.AMER |  | 4/60 | 6.7% |
| [98] | Schilling, 2012 | sheep | Germany | EU |  | 5/20 | 25.0% |
| [98] | Schilling, 2012 | goats | Germany | EU |  | 4/28 | 14.3% |

^a^source of animals not differentiated, all animals were farm animals in contact with the public.

^b^*Campylobacter upsaliensis* not differentiated in any of the studies, prevalence values represent all subtypes of *Campylobacter*
