# Supplementary material for: A Systematic Review and Meta-Analysis of the Campylobacter spp. Prevalence and Concentration in Household Pets and Petting Zoo Animals for Use in Exposure Assessments
Source: PLoS One. 2015 Dec 18;10(12):e0144976. doi: 10.1371/journal.pone.0144976 (PMC4684323; doi:10.1371/journal.pone.0144976)
Supplement: S4 Table — (DOCX) [file pone.0144976.s007.docx]

**S4 Table.**

| Ref. | First Author, Year | Animal | Country^a,b^ | Region^c,d,e^ | Diarrheic population | Sample source | Prevalence^f^ | % | Revised Prevalence^g^ | % |
| --- | --- | --- | --- | --- | --- | --- | --- | --- | --- | --- |
| [149] | Acke, 2009 | dogs | UK | EU | X | shelter | 19/22 | 86.4% | - | - |
| [149] | Acke, 2009 | dogs | UK | EU |  | shelter | 55/86 | 64.0% | - | - |
| [149] | Acke, 2009 | cats | UK | EU |  | shelter | 40/58 | 69.0% | - | - |
| [149] | Acke, 2009 | dogs | UK | EU |  | pets | 20/43 | 46.5% | - | - |
| [149] | Acke, 2009 | dogs | UK | EU | X | pets | 6/10 | 60.0% | - | - |
| [149] | Acke, 2009 | cats | UK | EU |  | pets | 11/25 | 44.0% | - | - |
| [149] | Acke, 2009 | cats | UK | EU | X | pets | 1/1 | 100.0% | - | - |
| [121] | Baker, 1999 | cats | Australia | AUS/NZ | X | shelter | 3/77 | 3.9% | 2/77 | 2.6% |
| [121] | Baker, 1999 | cats | Australia | AUS/NZ |  | shelter | 14/77 | 18.2% | 5/77 | 6.5% |
| [121] | Baker, 1999 | cats | Australia | AUS/NZ | X | kennel | 0/56 | 0.0% | - | - |
| [121] | Baker, 1999 | cats | Australia | AUS/NZ |  | kennel | 6/56 | 10.7% | 0/56 | 0.0% |
| [121] | Baker, 1999 | cats | Australia | AUS/NZ | X | clinic | 0/5 | 0.0% | - | - |
| [121] | Baker, 1999 | cats | Australia | AUS/NZ |  | clinic | 1/5 | 20.0% | 0/5 | 0.0% |
| [46] | Bender, 2005 | cats | USA | N.AMER | X^h^ | clinic, shelter | 8/152 | 5.3% | - | - |
| [90] | Burnens, 1992 | cats | Switzerland | EU | X | clinic | 21/156 | 13.5% | 10/156 | 6.4% |
| [90] | Burnens, 1992 | dogs | Switzerland | EU | X | clinic | 48/241 | 19.9% | 29/241 | 12.0% |
| [118] | Carbonero, 2002 | dogs | Spain | EU |  | clinic | 102/290 | 35.2% | 42/290 | 14.5% |
| [49] | Cave, 2002 | dogs | USA | N.AMER | X | clinic | 13/260 | 5.0% | 10/260 | 3.8% |
| [49] | Cave, 2002 | dogs | USA | N.AMER |  | clinic | 21/74 | 28.4% | - | - |
| [102] | Chaban, 2010 | dogs | Canada | N.AMER |  | pets | 39/70 | 55.7% | 9/70 | 12.9% |
| [102] | Chaban, 2010 | dogs | Canada | N.AMER | X | clinic | 63/65 | 96.9% | 8/65 | 12.3% |
| [114] | Engvall, 2003 | dogs | Sweden | EU |  | pets | 45/84 | 53.6% | - | - |
| [114] | Engvall, 2003 | dogs | Sweden | EU | X | pets | 6/7 | 85.7% | - | - |
| [74] | Gow, 2009 | cats | UK | EU |  | clinic | 5/57 | 8.8% | - | - |
| [88] | Guest, 2007 | dogs | UK | EU |  | kennel | 85/326 | 26.1% | - | - |
| [88] | Guest, 2007 | dogs | UK | EU |  | kennel | 24/117 | 20.5% | - | - |
| [88] | Guest, 2007 | dogs | UK | EU |  | pets | 16/106 | 15.1% | - | - |
| [88] | Guest, 2007 | dogs | UK | EU | X | pets | 18/59 | 30.5% | - | - |
| [62] | Hackett, 2003 | dogs | USA | N.AMER | X | clinic | 0/71 | 0.0% | - | - |
| [62] | Hackett, 2003 | dogs | USA | N.AMER |  | clinic | 1/59 | 1.7% | - | - |
| [92] | Hald, 1997 | cats | Denmark | EU |  | clinic | 2/42 | 4.8% | 0/42 | 0.0% |
| [92] | Hald, 1997 | dogs | Denmark | EU |  | clinic | 21/72 | 29.2% | 17/72 | 23.6% |
| [15] | Hald, 2004 | dogs | Denmark | EU |  | pets | 278/366 | 76.0% | 72/366 | 19.7% |
| [63] | Hill, 2000 | cats | USA | N.AMER | X | pets | 1/70 | 1.4% | - | - |
| [63] | Hill, 2000 | cats | USA | N.AMER |  | pets | 1/52 | 1.9% | - | - |
| [63] | Hill, 2000 | cats | USA | N.AMER | X | shelter | 0/12 | 0.0% | - | - |
| [63] | Hill, 2000 | cats | USA | N.AMER |  | shelter | 0/53 | 0.0% | - | - |
| [105] | Himsworth, 2010 | dogs | Canada | N.AMER |  | pets | 28/60 | 46.7% | 20/60 | 33.3% |
| [128] | Lee, 2004 | cats | USA | N.AMER |  | shelter | 0/27 | 0.0% | - | - |
| [128] | Lee, 2004 | cats | USA | N.AMER |  | pets | 0/10 | 0.0% | - | - |
| [128] | Lee, 2004 | dogs | USA | N.AMER |  | shelter | 10/55 | 18.2% | - | - |
| [128] | Lee, 2004 | dogs | USA | N.AMER |  | pets | 0/15 | 0.0% | - | - |
| [107] | Lefebvre. 2006 | dogs | Canada | N.AMER |  | pets | 0/102 | 0.0% | - | - |
| [150] | Lenz, 2009 | dogs | USA | N.AMER |  | pets | 1/42 | 2.4% | - | - |
| [150] | Lenz, 2009 | dogs | USA | N.AMER |  | pets | 0/49 | 0.0% | - | - |
| [108] | Leonard, 2011 | dogs | Canada | N.AMER |  | clinic | 6/240 | 2.5% | - | - |
| [151] | Marks, 2002 | dogs | USA | N.AMER |  | clinic | 29/58 | 50.0% | - | - |
| [151] | Marks, 2002 | dogs | USA | N.AMER |  | clinic | 13/42 | 31.0% | - | - |
| [151] | Marks, 2002 | dogs | USA | N.AMER | X | clinic | 7/32 | 21.9% | - | - |
| [87] | Moreno, 1993 | dogs | UK | EU |  | kennel | 25/56 | 44.6% | 21/56 | 37.5% |
| [87] | Moreno, 1993 | cats | UK | EU |  | kennel | 52/68 | 76.5% | 7/68 | 10.3% |
| [115] | Moser, 2001 | cats | Germany | EU | X | clinic | 5/13 | 38.5% | 4/13 | 30.8% |
| [115] | Moser, 2001 | cats | Germany | EU |  | clinic | 17/33 | 51.5% | 9/33 | 27.3% |
| [115] | Moser, 2001 | dogs | Germany | EU | X | clinic | 32/64 | 50.0% | 8/64 | 12.5% |
| [115] | Moser, 2001 | dogs | Germany | EU |  | clinic | 77/197 | 39.1% | 13/197 | 6.6% |
| [117] | Moyaert, 2008 | dogs | Belgium | EU | X | clinic | 21/37 | 56.8% | 8/37 | 21.6% |
| [117] | Moyaert, 2008 | dogs | Belgium | EU |  | clinic | 20/50 | 40.0% | 7/50 | 14.0% |
| [78] | Ogden, 2009 | cats | UK | EU |  | pets | 0/6 | 0.0% | - | - |
| [78] | Ogden, 2009 | cats | UK | EU |  | pets | 2/38 | 5.3% | - | - |
| [78] | Ogden, 2009 | dogs | UK | EU |  | pets | 0/11 | 0.0% | - | - |
| [78] | Ogden, 2009 | dogs | UK | EU |  | pets | 1/66 | 1.5% | - | - |
| [25] | Parsons, 2010 | dogs | UK | EU |  | clinic | 96/249 | 38.6% | 3/249 | 1.2% |
| [152] | Parsons, 2011 | dogs | UK | EU |  | shelter | 165/268 | 61.6% | 107/268 | 39.9% |
| [152] | Parsons, 2011 | dogs | UK | EU |  | kennel | 48/131 | 36.6% | 13/131 | 9.9% |
| [130] | Queen, 2012 | cats | USA | N.AMER | X | clinic, shelter | 21/219 | 9.6% | - |  |
| [130] | Queen, 2012 | cats | USA | N.AMER |  | pets | 15/54 | 27.8% | - |  |
| [116] | Rossi, 2008 | dogs | Italy | EU |  | clinic | 24/52 | 46.2% | 8/52 | 15.4% |
| [116] | Rossi, 2008 | dogs | Italy | EU | X | clinic | 29/138 | 21.0% | 12/138 | 8.7% |
| [116] | Rossi, 2008 | cats | Italy | EU |  | clinic | 8/21 | 38.1% | 5/21 | 23.8% |
| [116] | Rossi, 2008 | cats | Italy | EU | X | clinic | 19/63 | 30.2% | 16/63 | 25.4% |
| [99] | Sandberg, 2002 | dogs | Norway | EU | X | clinic | 18/66 | 27.3% | 3/66 | 4.5% |
| [99] | Sandberg, 2002 | dogs | Norway | EU |  | clinic | 124/529 | 23.4% | 22/159 | 13.8% |
| [99] | Sandberg, 2002 | cats | Norway | EU |  | clinic | 54/301 | 17.9% | 16/301 | 5.3% |
| [99] | Sandberg, 2002 | cats | Norway | EU | X | clinic | 5/31 | 16.1% | 1/31 | 3.2% |
| [24] | Sokolow, 2005 | dogs | USA | N.AMER |  | shelter | 16/60 | 16.7% | 10/60 | 26.7% |
| [24] | Sokolow, 2005 | dogs | USA | N.AMER | X | shelter | 31/60 | 51.7% | 24/60 | 40.0% |
| [129] | Spain, 2001 | cats | USA | N.AMER |  | pets | 2/114 | 1.8% | - | - |
| [89] | Stavisky, 2011 | dogs | UK | EU | X | clinic | 24/80 | 30.0% | - | - |
| [89] | Stavisky, 2011 | dogs | UK | EU |  | clinic | 63/147 | 42.9% | - | - |
| [153] | Steinhauserova I | cats | Czech Rep. | EU | X | clinic | 3/17 | 17.6% | - | - |
| [153] | Steinhauserova, 2000 | dogs | Czech Rep. | EU | X | clinic | 48/208 | 23.1% | 32/208 | 15.4% |
| [153] | Steinhauserova, 2000 | dogs and cats | Czech Rep. | EU |  | clinic | 19/126 | 15.1% | 16/126 | 12.7% |
| [119] | Westgarth, 2009 | dogs | UK | EU |  | pets | 48/183 | 26.2% | 2/183 | 1.1% |
| [154] | Wieland, 2005 | dogs | Switzerland | EU |  | clinic | 261/634 | 41.2% | 68/634 | 10.7% |
| [154] | Wieland, 2005 | cats | Switzerland | EU |  | clinic | 250/596 | 41.9% | 40/596 | 6.7% |

**^a^**UK = the United Kingdom

**^b^**USA = United States of America

**^c^**EU – Europe

**^d^**AUS/NZ = Australia or New Zealand

**^e^**N. AMER = Canada or the United States of America

^f^All values are fecal carriage

**^g^***Campylobacter upsaliensis* excluded when this data was available.

**^h^**Mixture of healthy and diarrheic animals.
